# Immune-checkpoint inhibitors for glioblastoma: what have we learned?

**DOI:** 10.1590/0004-282X-ANP-2022-S129

**Published:** 2022-08-12

**Authors:** Antonio Omuro

**Affiliations:** 1 Yale University, Yale School of Medicine, New Haven, USA.

**Keywords:** Glioblastoma, Immunotherapy, Immune Checkpoint Inhibitors, Nivolumab, Pembrolizumab, Glioblastoma, Imunoterapia, Inibidores de Checkpoint Imunológico, Nivolumabe, Pembrolizumabe

## Abstract

**Background:**

Glioblastoma, the most common malignant primary brain tumor, remains a lethal disease with few therapeutic options. Immunotherapies, particularly immune checkpoint inhibitors (ICPi), have revolutionized cancer treatment, but their role in glioblastoma is uncertain.

**Objective:**

To review the state of immunotherapies in glioblastoma, with an emphasis on recently published ICPi clinical trials.

**Methods:**

In this editorial/opinion article, we critically review results of the first generation of trials of ipilimumab, nivolumab and pembrolizumab in glioblastoma, as well as future directions.

**Results:**

Expression of PD-L1 is frequent in glioblastoma, ranging from 60-70% of patients. Phase 1 studies of nivolumab with and without ipilimumab, as well as pembrolizumab, showed no new safety concerns in brain tumors, and no neurotoxicity. However, randomized phase 3 trials of nivolumab showed no survival improvements over bevacizumab in recurrent glioblastoma; no role in newly diagnosed disease as a replacement for temozolomide in unmethylated MGMT promoter tumors; and no benefit as an addition to temozolomide in methylated MGMT tumors. However, studies examining post treatment tumor samples have shown signs of increased immunologic response, and occasional long lasting radiographic responses have been seen. A small study of pembrolizumab suggested a potential role as a “neoadjuvant” treatment in resectable recurrent glioblastoma, while other studies are investigating selection of patients with higher mutational burden and novel agents and combinatorial strategies.

**Conclusion:**

Despite initial negative trials, immunotherapy remains of high interest in glioblastoma, and many trials are still ongoing. Improving our mechanistic understanding of the immunosuppression and T cell dysfunction induced by both tumor and the CNS microenvironment remains however crucial for the development of successful immunotherapeutic approaches in this disease.

## RESUMO

**Antecedentes:**

Glioblastoma é o tumor cerebral primário maligno mais comum e continua sendo uma doença letal com poucas opções terapêuticas. As imunoterapias, em especial, os inibidores de checkpoint imunológico (ICPi), revolucionaram o tratamento do câncer, mas seu papel no glioblastoma é incerto.

**Objetivo:**

Revisar o estado atual do papel das imunoterapias no glioblastoma, com ênfase nos ensaios clínicos ICPi publicados recentemente.

**Métodos:**

Neste artigo de revisão, analisamos criticamente os resultados da primeira geração de estudos de ipilimumab, nivolumab e pembrolizumab em glioblastoma, bem como as perspectivas futuras.

**Resultados:**

A expressão de PD-L1 é frequente no glioblastoma, variando de 60-70% dos pacientes. Estudos de fase 1 de nivolumab com e sem ipilimumab, bem como pembrolizumab, não revelaram novas questões de tolerabilidade nem neurotoxicidade. No entanto, ensaios randomizados de fase 3 de nivolumab não apontaram melhorias na sobrevida em relação ao bevacizumab em glioblastoma recorrente, nenhum papel na doença recém-diagnosticada como substituto da temozolomida em tumores promotores de MGMT não metilados e nenhum benefício como adição à temozolomida em tumores MGMT metilados. No entanto, estudos que examinaram amostras de tumores pós-tratamento mostraram sinais de aumento da resposta imunológica, e respostas radiográficas ocasionais de longa duração foram observadas. Um pequeno estudo de pembrolizumab sugeriu um papel potencial como tratamento “neoadjuvante” no glioblastoma recorrente ressecável, ao passo que outros estudos estão investigando a seleção de pacientes com maior carga mutacional e novos agentes e estratégias combinatórias.

**Conclusão:**

Apesar dos ensaios iniciais negativos, a imunoterapia continua sendo de grande interesse no glioblastoma, e muitos ensaios ainda estão em andamento. No entanto, melhorar a nossa compreensão dos mecanismos de imunossupressão e disfunção das células T induzidas tanto pelo tumor quanto pelo microambiente do SNC continua sendo crucial para o desenvolvimento de abordagens imunoterapêuticas bem-sucedidas nesta doença.

Immunotherapies, such as immune checkpoint blockade, have revolutionized cancer treatment. In particular, the development of strategies targeting the CTLA-4 and the PD-1 pathways have resulted in significant improvements in OS (OS) in a variety of challenging cancers, including melanoma, non-small cell lung cancer, head and neck cancers and others. 

Immune checkpoints are regulatory inhibitory pathways involved in the maintenance of immunologic homeostasis by modulating intensity and duration of immune responses. Among other functions, such pathways prevent auto-immunity, maintain self-tolerance in physiologic conditions, and protect tissues from damage during infection^1^. Cancer often highjacks such pathways for evading immune system surveillance, a vulnerability that has been therapeutically exploited through the development of immune checkpoint inhibitors (ICPi). A plethora of drugs has been successfully tested and approved by the FDA in multiple indications. The use of drugs such as ipilimumab, nivolumab, pembrolizumab, atezolizumab, durvalumab, and other anti-PD-1 and PD-L1 inhibitors have since become widespread in oncology. However, certain cancers, particularly those with low mutational burden, have been found to be refractory to ICPi. 

Glioblastoma and other gliomas are highly attractive targets for immune checkpoint blockade^2^. Glioblastoma is the most common and aggressive form of primary brain tumor, and associated with dismal outcomes^3^. Treatment remains restricted to radiotherapy and alkylating agents, which seem to mostly benefit patients with tumors harboring methylated MGMT promoter, present in about a third of patients. Glioblastomas notoriously promote immunosuppression and may evade the immune system through multiple mechanisms. Implicated systemic factors include decreased T cell responsiveness, increase in Tregs, decreased monocyte and dendritic cell function, lower levels of immunoglobulins, frequent use of corticosteroids, and lymphopenia from treatments. Those add to unique local immunosuppressive factors such as down regulation of MHC molecules, secretion of inhibitory cytokines such as TGF-Beta, VEGF, PG-E2, IL-10, LLT-1, polarization of microglia and tumor associated macrophages towards the immunosuppressive M2 phenotype^4^, decreased T cell function due to hypoxia, T cell apoptosis through Fas, and, importantly, infiltration with Tregs and increased expression of immune checkpoints^5^. 

In 2017, we reported the first prospective clinical trial of ICPi in glioblastoma, which focused on anti-CTLA-4 and anti-PD-1 monoclonal antibodies^6^. That study was part of clinical trial Checkmate 143, which consisted of multiple cohorts across different lines of treatment for this disease, and sponsored by Bristol Myers Squib. In this phase 1 portion of the study, anti-PD-1 monoclonal antibody nivolumab was given with or without the anti-CTLA-4 monoclonal antibody ipilimumab to patients with recurrent disease. That study showed that the toxicity profile in this population was consistent with other cancers, and no new safety signals were identified. Importantly, there was no evidence of clinically significant neurotoxicity, which was a concern given the anatomical location in the brain. As expected, the combination of ipilimumab and nivolumab was more toxic than nivolumab alone, resulting in more frequent and more severe immune-related adverse events. PD-L-1 expression was high in archived tumor specimens from those patients (68%), and some signals of activity were observed, including a few radiographic responses and some patients displaying increased immune cells infiltrates on tissue biopsy^6^. 

Although phase 2 data was not available, several large trials of nivolumab were promptly launched in this disease, encouraged at the time by the excellent track record in other diseases with high frequency of PD-L1 expression, and the desperate need to rapidly develop novel therapies for glioblastoma. Because of a more favorable toxicity profile, and emerging evidence of efficacy as a single agent in other tumor types, nivolumab was initially selected for further development; accrual to these trials was fast, highlighting the interest from patients and physicians on this approach. 

The first randomized study was the phase 3 portion of Checkmate 143, which randomized recurrent glioblastoma patients to receive nivolumab or anti-VEGF therapy bevacizumab, a standard therapy for this disease setting^7^. Among the 369 randomized patients, the median OS was 9.8 months for the nivolumab arm and 10 months for bevacizumab (HR, 1.04; P = .76). Of note, there was no evidence of improved efficacy in PD-L1 expressing tumors. Interestingly, nivolumab-treated patients with MGMT-methylated tumors who had not received baseline corticosteroids seemed to experience prolonged survival in post-hoc analyses, although the study was underpowered to properly investigate that question. 

In the newly diagnosed setting, the first published study focused on the exploratory cohorts of CheckMate 143, which enrolled 136 patients^8^. In that study, the various cohorts investigated nivolumab added to radiotherapy and temozolomide. Given the lack of efficacy of temozolomide in this phenotype, nivolumab and radiotherapy were given without temozolomide in various cohorts of MGMT unmethylated patients. Overall, results demonstrated the feasibility of adding nivolumab to radiotherapy with or without temozolomide, with more toxicities observed when both temozolomide and nivolumab were given. The cohorts where temozolomide was omitted had lower incidence of lymphopenia, and survival results clearly varied according to MGMT methylation status. The median OS with nivolumab+RT+TMZ was 33 months in patients with methylated MGMT promoter, although that cohort was small (N=15). Across the different cohorts for MGMT unmethylated patients, median OS varied from 16.5 and 14.8 months for nivolumab+RT+TMZ and 14.4 and 14 months for nivolumab+RT. 

While results of Checkmate 143 were maturing, two randomized clinical trials were launched. Checkmate 498 focused on unmethylated MGMT newly diagnosed glioblastoma, and tested nivolumab as a potential replacement for temozolomide in this chemoresistant population (Omuro et al.^8^). Unfortunately, results showed that the addition of nivolumab to radiotherapy was actually inferior to chemotherapy with temozolomide. Among the 560 randomized patients, the median OS (mOS) was 13.4 months with NIVO+RT vs 14.9 months with TMZ+RT (HR, 1.31 [95% CI, 1.09-1.58]; *P*=.0037). Results seemed to reflect that temozolomide may have some efficacy in MGMT unmethylated patients as defined by the standard definitions and cutoffs for MGMT methylation. However, a nivolumab-related phenomenon of “hyperprogression” leading to inferior results cannot be entirely excluded, as has been suggested in other tumor types. Finally, Checkmate 548 was a randomized phase 3 study testing the addition of nivolumab to standard chemoradiotherapy in MGMT methylated glioblastoma, and similarly showed no improvements in OS (Weller et al, manuscript submitted). Of note, across all nivolumab studies, there was no correlation between PD-L1 expression and efficacy. Trials of the combination of nivolumab and ipilimumab are still ongoing.

Pembrolizumab is another anti-PD-1 antibody that has been tested in glioblastoma. In a phase 1 study, patients with glioblastoma expressing PD-L1 (>=1%) were eligible^9^. A total of 62% of screened patients displayed PD-L1 expressing tumors and a total of 26 patients were eventually enrolled. Again, only modest efficacy was observed, with ORR of 8%, with two partial responses observed. The 6-month progression-free survival was 37.7% and median OS was 13.1 months. In a randomized phase 2 study, pembrolizumab was given with and without bevacizumab, and achieved a median OS of 8.8 months (combination arm) and 10.3 months (pembrolizumab alone); authors concluded that both treatments were ineffective^10^. Of note, the unfavorable results of the combination arm damped the initial enthusiasm on the combination of ICPi with bevacizumab, which could potentially afford less corticosteroids usage and synergistic effects, but also decrease lymphocyte trafficking and cytokine release. Another phase 1 study^11^ investigated pembrolizumab with hypofractionated stereotactic re-irradiation and bevacizumab for recurrent glioblastoma and anaplastic astrocytoma, and found the combination to be feasible, with a median PFS of 8 months and OS of 13.5 months in bevacizumab naïve patients, which is difficult to interpret given the heterogeneity of the population enrolled. 

More interestingly, two small studies evaluated pembrolizumab in recurrent glioblastoma given as a “neoadjuvant therapy” prior to surgery, which allowed for analysis of post-treatment tumor specimens^12^ ^,^ ^13^. In both studies, an increase in T cell infiltration and antigen-reactive clonal expansion was observed in the post-treatment tumor microenvironment. One of those studies^12^ addressed the potential clinical benefit of the “neoadjuvant” usage, which has been suggested to provide an optimal setting for ICPi based on a priming effect that is magnified following tumor removal, as observed in other tumor types. In that study, 35 patients with recurrent, surgically resectable glioblastoma were randomized to receive neoadjuvant pembrolizumab, with continued adjuvant therapy following surgery (neoadjuvant arm), versus pembrolizumab given only after surgery without a neoadjuvant dose (adjuvant arm). The median OS was 13.7 m in the neoadjuvant arm, vs 7.5 m in the adjuvant arm (p=0.04). Moreover, neoadjuvant PD-1 blockade was associated with upregulation of T cell- and interferon-γ-related gene expression, downregulation of cell-cycle-related genes, focal induction of PD-L1 in the tumor microenvironment, enhanced clonal expansion of T cells, decreased PD-1 expression on peripheral blood T cells and decreasing monocytic population- all signs indicating improved immunological response. Another single arm trial of neoadjuvant pembrolizumab^14^ found an interesting OS of 20 months, although analysis of tumor tissue found a paucity of immune activation markers, and an abundance of CD68+ macrophages. Overall, the small sample sizes of studies to date preclude definitive conclusions on the clinical benefits of neoadjuvant ICPi, and further studies are needed to investigate this intriguing concept. 

Additional trials have investigated anti-PD-L1 antibody atezolizumab. In a small phase 1 trial, that drug was found to be safe, but efficacy was difficult to discern given the heavily pre-treated population enrolled^15^. Other trials are awaiting publication of results. 

Finally, some attempts have been made to identify optimal candidates for anti-PD-1 therapy. An exploratory retrospective study^16^ found enrichment of PTEN mutations associated with immunosuppressive expression signatures in tumors non-responsive to pembrolizumab or nivolumab, and an enrichment of MAPK pathway alterations (PTPN11, BRAF) in responders. Responsive tumors were also associated with branched patterns of evolution from the elimination of neoepitopes as well as with differences in T cell clonal diversity and tumor microenvironment profiles. However, these intriguing results are limited by the retrospective nature, the uncertain definition of responders vs non responders, and lack of adequate controls. 

**Figure 1.**
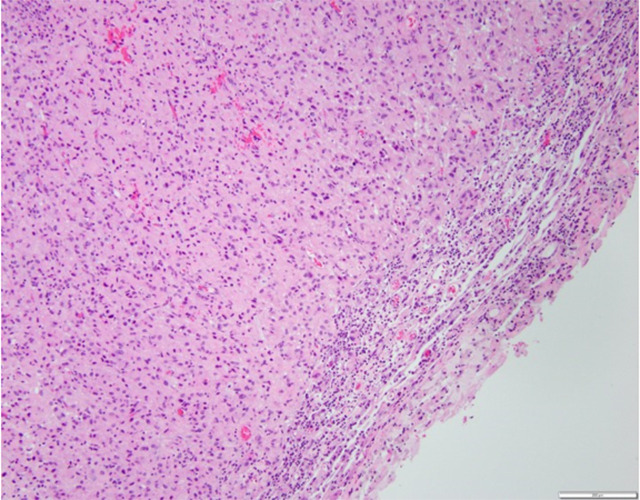
H/E staining of an MGMT unmethylated glioblastoma resected from a patient after exposure to anti-PD-1 therapy, showing intense inflammatory reaction and lymphocyte infiltration. Courtesy of Dr Marc Rosenblum.

In summary, experience with ICPi to date has overall yielded disappointing results. However, there is evidence of activity in select patients, in the form of radiographic responses, increased signs of immune response (Figure 1), and occasional patients achieving longer survival, perhaps with an advantage for the use in the neoadjuvant setting. Because immunotherapy is based on more stable targets than anti-tumor targeted therapies, this approach remains of high interest in Neuro-Oncology. Future directions include ongoing research to identify best candidates, while improving our mechanistic understanding of response and resistance to ICPi. At this time, it is unclear how much of the lack of activity derives from unique mechanisms pertaining to tumor growing in an immune privileged site such as the CNS, versus intrinsic mechanisms of resistance potentially shared with other solid tumors that are unresponsive to ICPi. Additional challenges posed by the location in the brain include the blood-brain barrier, which may limit leukocyte trafficking, as well as the unique anatomical and molecular specialization that allows only T cells that have been activated in the periphery to enter the CNS. T cells that populate the CNS have characteristics of tissue-resident memory T cells and are enriched for viral specificities; only limited antigen presentation in therefore possible in CNS tumors^5^. Indeed, the defective lymphatic drainage within the CNS has been found to be a crucial aspect of the poor T cell activation observed in glioblastoma models^17^. 

As for future directions, ongoing or planned trials are now seeking to investigate whether patients with increased tumor mutational burden and enhanced epitope landscapes^18^, such as gliomas with acquired or primary mismatch repair deficiency, could constitute better candidates for these therapies. Other trials are focusing on targeting new, alternative ICPi such as anti-TIGIT, LAG-3, CD137, TIM3 and other antibodies, both as single agents and in combinations. Other combinations being explored include the addition of alternative immune modulators and microenvironment modifiers, various types of vaccines, oncolytic viruses, epigenetic modifiers, chimeric antigen receptor T cells (CAR-T cells), as well as alternative treatment modalities such as hypofractionated radiotherapy and laser interstitial thermal therapy. However, success of such approaches remains contingent upon a better understanding and targeting of the mechanisms of T cell dysfunction and glioblastoma-associated immunosuppression, and of the unique barriers to successful immune responses posed by the CNS environment.
